# Lung Function Trajectories After Preterm Birth: A Life-Course Approach to Age-Specific Monitoring

**DOI:** 10.3390/children13040500

**Published:** 2026-04-02

**Authors:** Dorina Hoxha, Ilaria Bucci, Sabrina Di Pillo, Francesco Chiarelli, Marina Attanasi, Paola Di Filippo

**Affiliations:** Pediatric Allergy and Pulmonology Unit, Department of Pediatrics, University of Chieti-Pescara, Via dei Vestini n°5, 66100 Chieti, Italy; dorina.hoxha@studenti.unich.it (D.H.); ilaria.bucci@studenti.unich.it (I.B.); sabrina.dipillo@asl2bruzzo.it (S.D.P.); chiarelli@unich.it (F.C.); marina.attanasi@unich.it (M.A.)

**Keywords:** preterm birth, bronchopulmonary dysplasia, lung function, life-course respiratory outcomes, longitudinal lung function trajectories

## Abstract

Preterm birth interrupts critical phases of lung development and is associated with long-term alterations in respiratory structure and function. While bronchopulmonary dysplasia (BPD) has traditionally been considered the principal determinant of adverse outcomes, accumulating evidence indicates that prematurity per se contributes substantially to persistent pulmonary impairment. Lung function trajectories in preterm-born children frequently track along lower percentiles from infancy into adolescence and early adulthood, with limited catch-up growth and increased vulnerability to chronic airflow limitation. Assessment of lung function requires a developmentally tailored approach, as feasibility and interpretability vary across age groups. In infancy, non-volitional techniques such as tidal breathing flow-volume loop analysis and raised-volume rapid thoracoabdominal compression allow early evaluation of respiratory mechanics. During toddlerhood, methodological limitations persist, although emerging technologies may expand feasibility. In preschool children, impulse oscillometry enables detection of small airway dysfunction, often preceding spirometric abnormalities. From school age onward, spirometry, body plethysmography, diffusing capacity, and multiple breath washout provide complementary information on obstructive, restrictive, and gas-exchange impairments. Longitudinal studies demonstrate that reduced lung function is not confined to children with BPD and may predispose to early-onset chronic obstructive pulmonary disease-like phenotypes. Early identification of abnormal trajectories and modifiable risk factors supports structured long-term follow-up and preventive strategies. Standardization of age-specific assessment protocols and harmonization of reference values are essential to improve risk stratification and optimize long-term respiratory outcomes in this vulnerable population.

## 1. Introduction

**Figure 1:**
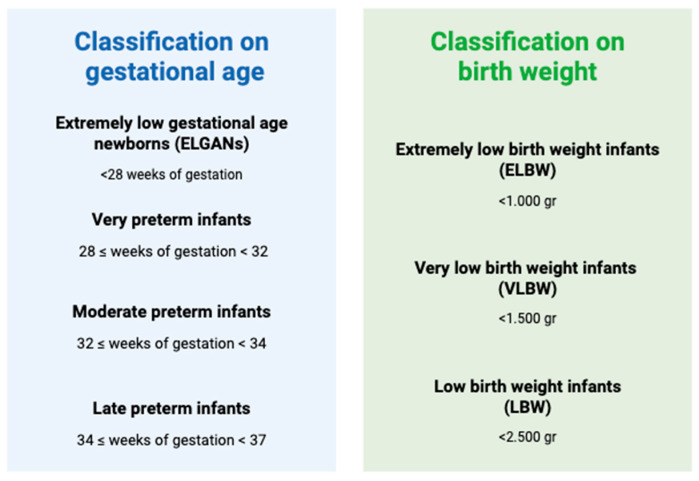
Classification of preterm infants according to gestational age and birth weight. Preterm neonates are categorized based on gestational age (extremely preterm, <28 weeks; very preterm, 28–31 weeks; moderate-to-late preterm, 32–36 weeks) and birth weight (extremely low birth weight, <1000 g; very low birth weight, <1500 g; low birth weight, <2500 g). (Created with BioRender.com).

Preterm birth affects approximately 6–14% of pregnancies worldwide and remains a major contributor to neonatal morbidity and long-term health burden [1,2]. Among its respiratory sequelae, bronchopulmonary dysplasia (BPD) represents the most recognized chronic complication and the most common chronic lung disease of infancy [3]. First described in 1967 in mechanically ventilated preterm neonates with severe hyaline membrane disease [4], BPD has undergone substantial clinical and pathological evolution. Advances in neonatal care (including antenatal corticosteroids, surfactant replacement therapy, and lung-protective ventilation strategies) have improved survival rates among extremely preterm infants [5]. In the surfactant era, the “new” BPD is characterized less by fibrosis and overt airway destruction and more by arrested alveolar and vascular development, resulting in simplified alveolar structures and dysmorphic pulmonary vasculature [6,7]. The incidence of BPD varies across regions and neonatal intensive care practices and is inversely related to gestational age and birth weight (Figure 1) [8,9].

Improved survival has led to a growing population of children at risk for persistent respiratory impairment. Importantly, adverse pulmonary outcomes are not limited to individuals with BPD. Prematurity itself, particularly when occurring during critical phases of alveolarization and angiogenesis, may permanently alter lung growth trajectories, predisposing to sustained airflow limitation, impaired gas exchange, and increased vulnerability to respiratory disease later in life [3,10]. Longitudinal studies indicate that many individuals born preterm track along lower lung function percentiles from early life and may fail to achieve optimal peak lung function in early adulthood, increasing susceptibility to early-onset chronic obstructive pulmonary disease (COPD)-like phenotypes, highlighting the importance of early identification and structured follow-up [11,12]. These observations challenge a dichotomous classification based solely on neonatal BPD status and instead support a broader continuum model of chronic lung disease of prematurity [11,13]. In this context, age-specific lung function assessment represents a critical tool for understanding the evolution of respiratory abnormalities across developmental stages. Because lung function testing feasibility and interpretation vary considerably from infancy to adolescence, a tailored and developmentally appropriate approach is required.

Accordingly, this narrative review is guided by the primary objective of synthesizing current evidence on lung growth and functional trajectories following preterm birth. Additionally, it aims to define the most informative age-specific monitoring strategies from infancy through adolescence, with a focus on clinical integration and long-term respiratory outcomes. More specifically, we address the following research question: which functional trajectories characterize individuals born preterm across development, and which age-based lung function tools are most appropriate for monitoring these trajectories in clinical practice? Addressing this question is clinically relevant because early recognition of unfavorable trajectories may support longitudinal surveillance, timely referral, and targeted preventive or therapeutic interventions.

## 2. Literature Search Methods

This review was developed as a narrative review and was structured in accordance with the Scale for the Assessment of Narrative Review Articles (SANRA) principles to enhance methodological transparency and interpretive rigor. SANRA principles guided the structured definition of the research question, transparency of literature selection, prioritization of clinically relevant sources, and balanced synthesis of evidence across developmental stages.

A comprehensive literature search was conducted in Pub-Med/MEDLINE, Scopus, Web of Science, and the Cochrane Library for studies published from January 2010 to January 2026. To provide a comprehensive historical perspective on bronchopulmonary dysplasia and infant lung function testing, seminal older papers were also included when deemed necessary. Search terms and MeSH headings included combinations of “bronchopulmonary dysplasia”, “prematurity”, “preterm birth”, “lung function”, “spirometry”, “oscillometry”, “impulse oscillometry”, “multiple breath washout”, “lung clearance index”, “tidal breathing”, “infant lung function”, “diffusing capacity”, and “longitudinal respiratory outcomes”.

Representative Boolean combinations included “preterm birth” AND “lung function” AND “spirometry” AND “bronchopulmonary dysplasia”.

Inclusion criteria:

- Human studies involving preterm-born infants, children, adolescents, or adults with outcomes relevant to lung development or lung function after prematurity.
- Longitudinal cohorts, observational studies, systematic reviews, meta-analyses, clinical guidelines, and technical standards.
- Studies reporting age-specific lung function tools, physiological parameters, longitudinal trajectories, or long-term respiratory outcomes.
- Articles published in English.

Exclusion criteria:

- Animal studies and non-physiological experimental models.
- Single case reports.
- Studies not specifically addressing pulmonary function trajectories, age-specific monitoring tools, or long-term respiratory outcomes after prematurity.

Additionally, the reference lists of key articles were manually screened to identify further relevant studies.

## 3. Disrupted Lung Development After Preterm Birth

### 3.1. Normal Lung Development and Timing of Preterm Birth

Normal lung development is a highly coordinated and tightly regulated process that begins in early embryogenesis and continues well into childhood [14,15]. It is traditionally divided into five stages: embryonic, pseudoglandular, canalicular, saccular, and alveolar [14]. The embryonic and pseudoglandular stages establish the bronchial tree through branching morphogenesis, whereas the canalicular stage (16–26 weeks of gestation) marks the differentiation of type I and type II pneumocytes and the initial formation of the air-blood barrier [16]. During the saccular stage (approximately 26–36 weeks), terminal saccules form, the interstitium thins, and capillary networks expand in preparation for postnatal gas exchange [17]. True alveolarization begins late in gestation and continues postnatally, extending into early childhood, with ongoing septation and expansion of the gas-exchange surface [15]. Extremely and very preterm birth (<28–32 weeks of gestation) therefore occurs during the canalicular and early saccular phases, when alveolar and vascular development are incomplete. Infants born at these stages have structurally immature lungs with reduced gas-exchange capacity and simplified architecture. Because alveolarization and pulmonary vascular growth continue after birth into infancy and childhood, postnatal environmental exposures, rather than intrauterine programming alone, play a pivotal role in shaping subsequent lung growth trajectories [18].

### 3.2. Pathophysiology of the “New Bronchopulmonary Dysplasia”

BPD is now recognized as a developmental lung disorder rather than solely a consequence of ventilator-induced injury. The “new” BPD is characterized primarily by arrested alveolarization and impaired pulmonary vascular development [6,7]. Preterm birth exposes the immature lung to mechanical ventilation, oxygen toxicity, infection, inflammation, and nutritional deficits [19,20,21,22]. Oxidative stress and inflammatory cascades disrupt signaling pathways essential for septation and angiogenesis, leading to simplified alveoli, reduced capillary density, and abnormal vascular remodeling [23,24,25]. The resulting phenotype is characterized by fewer and larger alveoli, decreased gas-exchange surface area, and dysmorphic pulmonary vasculature.

Hyperoxia following birth and intermittent hypoxia related to apnea further amplify oxidative and inflammatory injury [20,26]. These mechanisms affect not only parenchymal development but also airway growth and smooth muscle regulation, predisposing to later airflow limitation [27]. Vascular disruption is increasingly recognized as central to BPD pathogenesis and may underlie long-term reductions in diffusing capacity and cardiopulmonary reserve [23,24,25].

An additional consideration is that many contemporary extremely preterm infants are now exposed to neonatal care strategies that differ substantially from those of historical cohorts. These strategies include early continuous positive airway pressure [28], selective surfactant administration, less-invasive surfactant administration or minimally invasive surfactant therapy (LISA/MIST), and reduced duration of invasive ventilation [29]. Although long-term follow-up data from these newer cohorts are still emerging, available evidence suggests that respiratory trajectories may be somewhat attenuated compared with the classic BPD era, while not being completely normalized. Accordingly, interpretation of lung function outcomes should account for birth era and neonatal management, as contemporary cohorts may show subtler but still clinically relevant abnormalities in airflow, ventilation inhomogeneity, and gas-exchange capacity [30].

### 3.3. Prematurity Beyond BPD: A Broader Developmental Paradigm

Although BPD represents the most severe expression of neonatal lung injury, growing evidence indicates that prematurity itself (independent of a formal BPD diagnosis) can alter lung growth trajectories [3]. Even moderate-to-late preterm infants may exhibit subtle but persistent impairments in airflow and gas exchange compared with term-born peers [18]. These findings support a continuum model of chronic lung disease of prematurity, encompassing a spectrum of structural and functional alterations with heterogeneous clinical expression [11,13]. Rather than reflecting a binary distinction based on neonatal oxygen requirement, long-term respiratory outcomes likely result from the interaction between disrupted developmental programming and postnatal environmental exposures. From a functional perspective, arrested alveolarization and dysregulated vascular development may translate into persistent airflow obstruction, ventilation inhomogeneity, reduced diffusing capacity, and impaired cardiopulmonary reserve. These pathophysiological alterations form the biological substrate underlying the abnormal lung function trajectories observed from infancy through adulthood [3,10,11].

## 4. Lung Function Phenotypes After Preterm Birth

Preterm birth is associated with heterogeneous patterns of pulmonary dysfunction that may persist into adulthood. Rather than representing a single uniform phenotype, respiratory sequelae after prematurity encompass a spectrum of obstructive, restrictive, and mixed abnormalities, often reflecting the degree of developmental disruption and postnatal injury [11,31].

### 4.1. Persistent Airflow Obstruction

The predominant functional pattern described across cohorts of children and adolescents born preterm is airflow limitation. Reduced forced expiratory volume in one second (FEV_1_), lower FEV_1_/forced vital capacity (FVC) ratio (FEV_1_/FVC), and decreased forced expiratory flow between 25% and 75% of FVC (FEF25–75) have been consistently reported compared with term-born peers [31]. Although impairment is often more pronounced in individuals with a history of BPD, reduced spirometric indices are also observed in those without BPD, underscoring the contribution of prematurity itself to obstructive lung disease [10,11]. Unlike classical atopic asthma, prematurity-associated obstructive lung disease frequently shows limited eosinophilic inflammation and variable bronchodilator responsiveness, suggesting mechanisms related to impaired airway development rather than primary allergic inflammation [10,32].

### 4.2. Peripheral Airway Dysfunction and Ventilation Inhomogeneity

Small airway involvement represents a central feature of lung disease following preterm birth. Techniques sensitive to distal airway function, including impulse oscillometry (IOS) and multiple breath washout, frequently reveal abnormalities even when conventional spirometry is within normal limits [33,34].

Increased frequency dependence of resistance (R5–R20), abnormal reactance values, and elevated lung clearance index indicate ventilation inhomogeneity and peripheral airway dysfunction. These findings support the hypothesis that early disruption of airway growth and alveolarization leads to persistent small airway vulnerability [10,34].

### 4.3. Impaired Gas Exchange and Vascular Sequelae

Beyond airflow limitation, reduced diffusing capacity for carbon monoxide (DLCO) has been reported in school-age children and adolescents born extremely or very preterm [10]. This finding suggests persistent abnormalities at the alveolar-capillary interface consistent with underlying parenchymal and vascular developmental impairment [3,25]. Notably, such impairments may be present even in individuals without BPD [10].

### 4.4. Restrictive and Mixed Patterns

Although obstruction predominates, restrictive or mixed ventilatory defects have also been described, particularly following severe neonatal lung injury [11,35]. Reduced total lung capacity (TLC) and elevated residual volume-to-total lung capacity ratios (RV/TLC) indicate altered lung growth and air trapping, reflecting combined parenchymal and airway involvement [35,36,37].

### 4.5. From Phenotype to Trajectory

These phenotypes are not static. Longitudinal data indicate that many individuals born preterm follow persistently reduced lung function trajectories and may fail to achieve optimal peak lung function in early adulthood [11,12]. This developmental pattern may increase vulnerability to COPD-like phenotypes, particularly in the presence of additional environmental exposures [11]. Collectively, these phenotypes reflect the functional expression of disrupted lung development and form the clinical substrate that underlies age-specific monitoring strategies.

## 5. Age-Specific Lung Function Assessment

To improve practical applicability, we also propose a pragmatic follow-up pathway based on developmental stage, test feasibility, symptom burden, and prior results, acknowledging that this pathway is intended as a clinically oriented synthesis rather than a formal guideline.

Assessment of lung function in preterm-born children requires a developmentally tailored approach, as feasibility, methodological reliability, and interpretability vary substantially across age groups. Because structural and functional abnormalities may evolve over time, serial evaluation using age-appropriate techniques is essential to characterize respiratory trajectories and inform clinical management [38].

**Figure 2:**
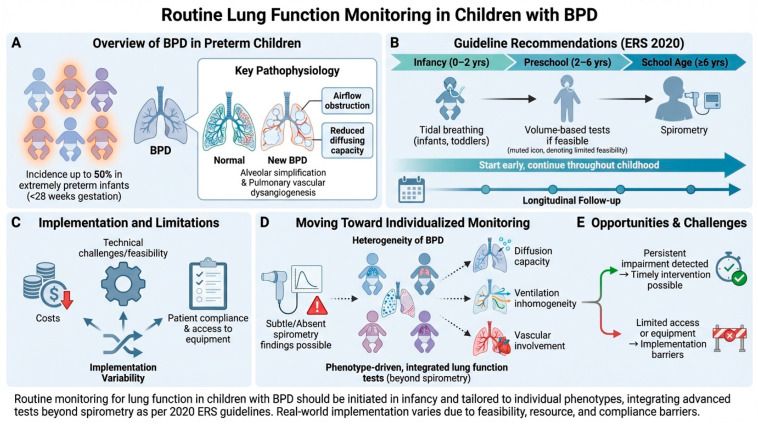
Routine lung function monitoring in children with bronchopulmonary dysplasia. Schematic overview of structured pulmonary follow-up in children with BPD. (**A**) Epidemiology and key pathophysiological features, including alveolar simplification, pulmonary vascular dysanapsis, airflow obstruction, and reduced diffusing capacity. (**B**) European Respiratory Society (ERS) 2020 guideline recommendations for age-specific monitoring, emphasizing early initiation in infancy, longitudinal follow-up, and transition from tidal breathing techniques in early life to spirometry from school age. (**C**) Practical implementation challenges, including technical feasibility, costs, and patient adherence. (**D**) The need for phenotype-driven assessment integrating advanced tests beyond spirometry to capture ventilation inhomogeneity, diffusion impairment, and vascular involvement. (**E**) Clinical implications, highlighting opportunities for early detection and timely intervention, alongside real-world barriers to implementation. (Created with FigureLabs, https://www.figurelabs.ai/?utm_source=google&utm_medium=cpc&utm_campaign=pmax_xhs&gad_source=1&gad_campaignid=23643798701&gclid=CjwKCAjwhLPOBhBiEiwA8_wJHDdu06tov0Su7Zui_ItbuNjPV0twwPw8xLM6GeJIVMU30JPr_BbivRoCVOMQAvD_BwE, accessed on 2 February 2026).

Despite this, implementation remains inconsistent across centers, largely due to technical challenges and limited availability of specialized equipment, particularly in infancy and early childhood. The 2020 European Respiratory Society (ERS) guidelines provide recommendations for systematic lung function monitoring in children with BPD (Figure 2). These recommendations are based on systematic evidence reviews evaluating age-specific assessment strategies, from tidal breathing techniques in infants and toddlers to spirometry in school age children. They also have considered feasibility, cost, and patient adherence [38]. An overview of age-specific lung function techniques, principal physiological parameters, methodological strengths and limitations, and evidence of abnormalities reported in preterm-born populations is shown in Table 1.

### 5.1. Infancy (0–12 Months)

In infancy, lung function assessment relies on non-volitional techniques performed during quiet sleep or under mild sedation [39]. These approaches primarily evaluate passive respiratory mechanics and early airflow dynamics. Tidal breathing flow-volume loops (TBFVL) represent one of the most feasible approaches in the first months of life, as measurements can be obtained during natural sleep without active cooperation [39]. Parameters such as tidal volume corrected for body weight (VT/kg), respiratory rate (RR), the ratio of time to peak tidal expiratory flow to total expiratory time (tPTEF/tE) and the ratio of volume to peak tidal expiratory flow to expiratory volume (VPTEF/VE) have been shown to differentiate infants with and without BPD [30]. Reduced tPTEF/tE ratios (commonly below 0.25–0.30) and abnormal expiratory loop morphology are considered markers of early airflow limitation and altered respiratory mechanics [40]. Infants with BPD typically exhibit lower VT/kg and tPTEF/tE values together with higher RR compared with those without BPD. Morphological abnormalities of the expiratory loop, including concavity, prolonged expiration, and reduced tidal flows, further reflect heterogeneous airway narrowing and impaired elastic recoil [40]. Early abnormalities in tidal breathing parameters have also been associated with increased respiratory morbidity later in childhood [18,41]. The infant’s arousal state significantly influences TBFVL measurements. Values obtained during wakefulness are characterized by higher tPTEF/tE and RR compared with sleep, whereas tidal volume remains relatively stable. These findings underscore the importance of arousal-state-specific normative values, particularly when sleep measurements are not feasible [42].

Prenatal and environmental exposures may further modulate early lung function. Maternal smoking during pregnancy has been associated with lower tidal volumes and increased lung clearance index, while in utero alcohol exposure and household benzene exposure have been linked to reduced tPTEF/tE ratios, highlighting the impact of modifiable environmental factors on early lung development [43]. Raised-volume rapid thoracoabdominal compression (RVRTC) enables the measurement of forced expiratory flows in infants, including maximal expiratory flow at functional residual capacity (VmaxFRC) [44,45]. However, the need for sedation, specialized equipment, and technical expertise limits its use primarily to research settings and tertiary referral centers.

### 5.2. Toddlerhood (1–3 Years)

Toddlerhood represents a particularly challenging and often underserved window for lung function assessment. This period coincides with rapid postnatal lung growth, ongoing alveolarization, and airway remodeling, representing a critical phase for identifying evolving respiratory abnormalities. However, limited cooperation and the progressive transition from infant-specific non-volitional techniques to preschool methodologies complicate standardized evaluation. In addition, the scarcity of commercially available devices specifically designed for this intermediate age group creates a substantial gap in systematic respiratory monitoring during a vulnerable stage of lung development [46].

Emerging technologies may help bridge this gap by enabling the collection of physiologically meaningful respiratory data without requiring forced maneuvers. Ultrasound-based spirometry systems integrating tidal-breathing modules allow acquisition of resting flow-volume signals in children unable to perform reproducible forced efforts. The addition of capnography permits assessment of ventilation efficiency and physiological dead space. However, normative reference values and clinical validation in ex-preterm toddlers and survivors of BPD remain limited, and their role is currently confined mainly to structured follow-up programs [47].

Impedance pneumography-based tidal breathing analysis has demonstrated feasibility from approximately 12 months of age, enabling measurement of indices such as the expiratory variability index during overnight sleep and potentially supporting home-based monitoring. Nevertheless, its prognostic value in preterm-born populations has yet to be fully established [48].

Structured light plethysmography offers a non-contact assessment of thoracoabdominal motion and may be suitable for repeated measurements in young children. However, validation data in ex-preterm cohorts are currently scarce, and standardized interpretation frameworks are lacking [49]. Overall, despite technological advances, lung function assessment in toddlers born preterm remains constrained by methodological heterogeneity and insufficient validation. Development of standardized protocols and age-specific reference values is essential for broader clinical integration.

In standard clinical settings with limited advanced tools, current practical management of toddlers relies on structured clinical follow-up rather than routine instrumental testing. This includes periodic assessment of respiratory symptoms, exacerbation frequency, oxygen requirement, medication use, growth, feeding tolerance, and physical activity, with particular attention to symptom persistence between viral infections. Clinical monitoring alone may be sufficient in stable toddlers, but earlier referral to a specialized respiratory center should be considered in the presence of red flags such as persistent tachypnea, recurrent wheeze or hospitalizations, failure to thrive, exercise or feeding intolerance, resting or sleep-related desaturation, suspected pulmonary hypertension, or discordance between symptom burden and routine clinical findings [30,38]. 

### 5.3. Preschool Age (3–6 Years)

IOS has markedly expanded lung function assessment in preschool-aged children by enabling evaluation during quiet tidal breathing without the need for forced expiratory maneuvers [50,51]. This characteristic makes IOS particularly suitable for children unable to perform reliable and reproducible spirometric maneuvers.

IOS applies low-amplitude pressure oscillations at multiple frequencies (typically 5–35 Hz) during spontaneous breathing and analyzes the resulting pressure-flow relationship to derive respiratory system impedance, which includes resistance and reactance components. Resistance at 5 Hz (R5) reflects total airway resistance, whereas resistance at 20 Hz (R20) predominantly represents central airway resistance. The frequency-dependent difference between R5 and R20 (R5–R20) is commonly used as a surrogate marker of peripheral airway dysfunction. Reactance at 5 Hz (X5), along with derived parameters such as resonant frequency and reactance area, provides insight into distal airway and lung tissue mechanics [50,51].

Technical standards have emphasized the importance of equipment standardization and quality control to ensure reproducibility and comparability across devices [51]. Nevertheless, interpretation remains influenced by inter-device variability and the limited availability of robust, population-specific reference equations [52,53].

In preterm-born children, IOS frequently identifies abnormalities consistent with persistent small airway dysfunction extending into preschool and school age [10,33]. Increased R5 and R5–R20, more negative X5 values, and elevated reactance area support the concept of distal airway vulnerability following disrupted lung development. Although impairments are often more pronounced in individuals with extreme prematurity or a history of BPD, several contemporary cohorts have reported overlapping IOS profiles between children with and without BPD, underscoring the heterogeneity of outcomes and the contribution of prematurity per se [33,54].

Importantly, the oscillometric pattern observed in preterm-born children differs from that typically described in atopic asthma. Children with prematurity-associated obstructive lung disease (POLD) exhibit greater overall impedance and altered intra-breath oscillatory dynamics compared with term-born controls, suggesting that airflow limitation in this population reflects developmental airway and parenchymal alterations rather than predominantly inflammatory mechanisms [31,54].

Overall, IOS provides a sensitive tool for detecting early peripheral airway dysfunction in preterm-born children and complements spirometry by identifying abnormalities that may precede overt changes in conventional indices.

### 5.4. School Age and Adolescence

From approximately 5–6 years of age, conventional spirometry becomes feasible and represents the cornerstone of lung function evaluation in preterm-born children [55]. When performed according to the 2019 American Thoracic Society (ATS)/ERS technical standards (including appropriate calibration, infection control measures, and rigorous quality control) spirometry provides reproducible and clinically meaningful assessment of airflow limitation [55].

Key parameters derived from volume-time and flow-volume curves include FEV_1_, FVC, FEV_1_/FVC ratio, peak expiratory flow (PEF), and mid-expiratory flows such as FEF25–75. Results should be expressed as z-scores using Global Lung Function Initiative reference equations and interpreted relative to the lower limit of normal, allowing standardized comparison across age, sex, height, and ethnicity [55,56].

Large cohort studies consistently demonstrate persistent reductions in spirometric indices among children and adolescents born preterm compared with term-born peers [11,31]. The predominant functional phenotype is obstructive, characterized by reduced FEV_1_, lower FEV_1_/FVC ratio, and diminished mid-expiratory flows, suggesting ongoing small airway involvement. Importantly, these abnormalities are observed not only in survivors of bronchopulmonary dysplasia (BPD) but also in preterm-born individuals without a formal neonatal BPD diagnosis [10,57], underscoring the impact of prematurity itself on long-term respiratory outcomes.

This is clinically relevant because ventilation inhomogeneity may persist in school-aged children born very preterm even when spirometric indices remain within the normal range. In this context, an elevated LCI can uncover subtle peripheral airway disease earlier than conventional spirometry and may therefore complement follow-up in children with persistent symptoms, discordant clinical and spirometric findings, or suspected small-airway involvement [58].

While spirometry allows functional pattern recognition, it does not establish etiological diagnosis. Clinically, many BPD survivors present with mild-to-moderate airflow obstruction and incomplete catch-up growth, whereas more severe phenotypes may show restrictive or mixed defects consistent with impaired alveolar and somatic lung development. Longitudinal evidence suggests that failure to achieve optimal peak lung function during adolescence may predispose to chronic obstructive pulmonary disease-like trajectories in early adulthood [11,13].

Beyond spirometry, advanced lung function tests provide complementary insights into lung mechanics and gas exchange, allowing a more comprehensive characterization of respiratory sequelae in ex-preterm children.

Body plethysmography enables measurement of static lung volumes, including total lung capacity (TLC), residual volume (RV), and functional residual capacity (FRC), facilitating differentiation between obstructive, restrictive, and mixed ventilatory defects [59,60,61]. Increased RV and elevated RV/TLC ratios, frequently reported in preterm-born cohorts, reflect air trapping and altered airway mechanics and may be present even when spirometric indices appear within normal limits [35,36,37].

Measurement of diffusing capacity of the lung for carbon monoxide (DLCO) provides insight into alveolar-capillary membrane integrity and effective gas-exchange surface area, identifying abnormalities not detectable by spirometry alone [62]. Reduced DLCO has been described in school age children and adolescents born extremely or very preterm, independent of BPD status [10]. DLCO values correlate positively with gestational age, suggesting a developmental gradient in gas-exchange capacity. Notably, these impairments often occur in the absence of eosinophilic inflammation or elevated exhaled nitric oxide, supporting a non-eosinophilic mechanism and reinforcing the distinction between prematurity-associated lung disease and classical asthma [10]. Multiple breath washout testing further enhances detection of peripheral airway dysfunction by quantifying ventilation inhomogeneity through the lung clearance index. Elevated lung clearance index values have been reported in preterm-born children, indicating persistent small airway abnormalities not always captured by spirometry [34]. However, the long-term prognostic implications of abnormal multiple breath washout findings remain under investigation.

## 6. Longitudinal Lung Function Trajectories and Risk of Early Chronic Lung Disease

Preterm birth is increasingly recognized not only as a neonatal condition but as a determinant of lifelong respiratory health. Lung function development follows characteristic trajectories from infancy to adulthood, with peak lung function typically achieved in early adulthood. Disruption of early lung growth may result in persistently reduced trajectories, even in the absence of progressive disease [11,12].

### 6.1. Tracking of Lung Function from Childhood to Adulthood

Longitudinal cohort studies consistently demonstrate that preterm-born children follow lower lung function trajectories than term-born peers, with many failing to achieve full catch-up growth during adolescence and early adulthood. This pattern is characterized by persistently reduced FEV_1_ and FEV_1_/FVC ratios, reflecting sustained airflow limitation across developmental stages [11,12].

Lung function tracking appears to be established early in life. Infants and preschool children with impaired functional indices are more likely to exhibit reduced spirometric values at school age, suggesting that early structural and physiological alterations define subsequent respiratory capacity [12,18]. Early markers (including reduced tPTEF/tE ratios in infancy, elevated IOS resistance values, and lower FEV_1_ in later childhood) have been associated with ongoing obstruction, recurrent respiratory symptoms, and reduced exercise tolerance [12].

Long-term follow-up data further indicate that early airflow limitation may persist or even progress over time, particularly in individuals with severe BPD. Bergström et al. [63] reported a longitudinal decline in FEV_1_/FVC ratio in children with severe BPD, consistent with evolving chronic airflow limitation, while peripheral airway dysfunction assessed by IOS remained detectable across age groups. Similarly, Gray et al. [41] demonstrated that lower lung function at 1 year of age independently predicted increased respiratory symptoms and hospitalizations throughout early childhood, even after adjustment for neonatal measurements, underscoring the prognostic value of serial assessment rather than single time-point evaluation.

Although BPD has traditionally been viewed as the principal determinant of adverse outcomes, contemporary evidence indicates that prematurity itself substantially influences long-term lung function [10,57]. While impairment is often more pronounced in moderate-to-severe BPD, reduced spirometric and diffusing capacity indices have also been documented in preterm-born individuals without BPD [11]. These findings support a continuum model of chronic lung disease of prematurity rather than a dichotomous classification based solely on neonatal oxygen requirement.

**Figure 3:**
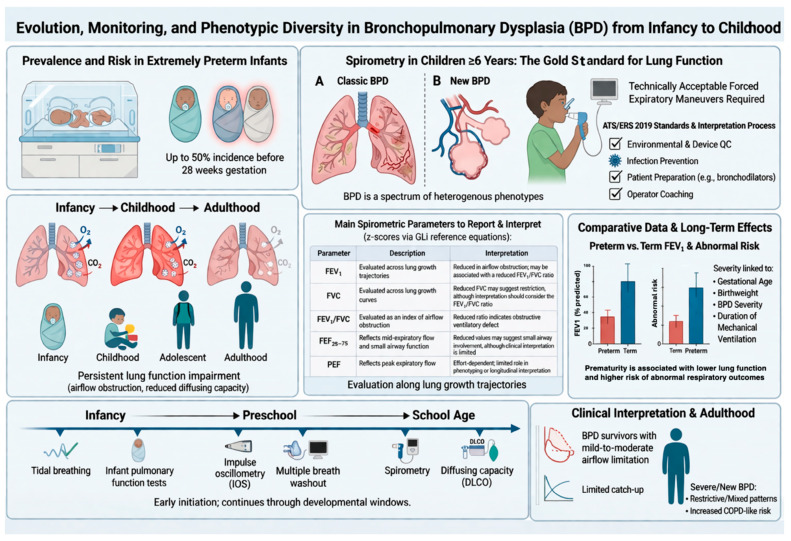
Evolution, monitoring, and phenotypic diversity of bronchopulmonary dysplasia from infancy to adulthood. Graphical overview of the prevalence, developmental trajectory, functional assessment, and long-term consequences of BPD. The upper panels illustrate the high incidence of BPD in extremely preterm infants (<28 weeks’ gestation) and the spectrum of classic and “new” BPD phenotypes. The central panels summarize key spirometric parameters to report and interpret (FEV_1_, FVC, FEV_1_/FVC, FEF25–75, PEF), expressed as z-scores according to GLI reference equations, and highlight differences between preterm- and term-born individuals. The lower panels depict age-specific lung function assessment from infancy (tidal breathing techniques and infant lung function testing) to preschool (impulse oscillometry, multiple breath washout) and school age (spirometry and diffusing capacity), emphasizing longitudinal tracking of lung function and the risk of persistent airflow limitation and reduced diffusing capacity into adolescence and adulthood. Clinical implications, including limited catch-up growth and increased susceptibility to COPD-like phenotypes, are also illustrated. BPD: bronchopulmonary dysplasia; ATS/ERS: American Thoracic Society/European Respiratory Society; QC: quality control; GLI: Global Lung Function Initiative; FEV_1_: forced expiratory volume in 1 s; FVC: forced vital capacity; FEV_1_/FVC: ratio of forced expiratory volume in 1 s to forced vital capacity; FEF25–75: forced expiratory flow between 25% and 75% of FVC; PEF: peak expiratory flow; IOS: impulse oscillometry (system); DLCO: diffusing capacity of the lung for carbon monoxide; TLC: total lung capacity; COPD: chronic obstructive pulmonary disease. (Created with FigureLabs).

Failure to achieve optimal peak lung function during adolescence may increase susceptibility to early-onset COPD-like phenotypes in adulthood, particularly when additional environmental insults are present (Figure 3) [11,12].

From a clinical standpoint, distinguishing prematurity-associated obstructive lung disease from childhood asthma remains challenging. Former preterm children may exhibit wheeze, exercise limitation, or bronchodilator responsiveness, yet often lack the atopic background, eosinophilic inflammation, or raised exhaled nitric oxide that typically support an asthma phenotype. Importantly, prematurity-associated obstructive lung disease (POLD) should be distinguished from classical childhood asthma. Although some overlap exists in clinical presentation and bronchodilator responsiveness, POLD is typically characterized by non-eosinophilic mechanisms, limited atopy, and structural airway and parenchymal development rather than predominantly inflammatory pathways. This distinction has important implications for treatment strategies and long-term management. Consequently, careful phenotyping is essential to avoid misclassification and inappropriate treatment [10].

This distinction is important because bronchodilator responsiveness does not necessarily imply classic type-2 airway inflammation, and therapeutic decisions should therefore be individualized, integrating symptom pattern, inflammatory markers when available, longitudinal lung function, and response to carefully monitored treatment trials [64].

### 6.2. Risk of Early-Onset Chronic Obstructive Lung Disease

Failure to achieve optimal peak lung function during adolescence is a recognized risk factor for the development of COPD later in life. Individuals born extremely or very preterm may enter adulthood with reduced pulmonary reserve, potentially increasing vulnerability to additional environmental insults such as tobacco smoke exposure, air pollution, or recurrent infections [11,13].

Although most preterm-born young adults do not meet formal diagnostic criteria for COPD, spirometric patterns characterized by mild airflow obstruction and reduced diffusing capacity have been described. This phenotype has been termed POLD, underscoring its developmental rather than predominantly inflammatory origin [32,54]. Long-term follow-up studies suggest that a subset of individuals may experience accelerated decline superimposed on an already reduced baseline, raising concerns about premature respiratory aging [12].

Emerging interventional data suggest that aspects of this impairment may remain biologically active and potentially modifiable. Course et al. [65] demonstrated that reduced desmosomal protein expression in exhaled breath condensate of school age children with BPD and impaired lung function normalized following treatment with inhaled corticosteroids and long-acting β_2_-agonists, supporting the hypothesis that ongoing airway remodeling processes may respond to targeted therapies.

### 6.3. Modifying Factors and Clinical Implications

Lung function trajectories after preterm birth are influenced by multiple interacting determinants, including degree of prematurity and birth weight, severity of neonatal lung injury, postnatal respiratory infections, environmental exposures such as tobacco smoke, and growth and nutritional status [10,11,12]. However, not all neonatal respiratory interventions appear to independently determine long-term functional impairment, as short-term invasive mechanical ventilation has not consistently been associated with reduced lung function in later childhood [66]. These factors operate across developmental stages and contribute to the marked heterogeneity observed in long-term respiratory outcomes.

Identification of modifiable risk factors is essential to inform preventive strategies and optimize long-term pulmonary health [11]. Recognition of persistently reduced lung function trajectories supports the need for structured follow-up extending beyond early childhood, as recommended by international guidelines [38]. Early identification of individuals at risk enables targeted counseling, minimization of environmental exposures, optimized vaccination strategies, and individualized therapeutic approaches [11,12].

Rather than focusing exclusively on the presence or absence of BPD, clinicians should integrate gestational age, early lung function indices, and longitudinal trends when assessing long-term respiratory risk [10,57].

## 7. Practical Considerations for Clinical Follow-Up

### 7.1. Age-Appropriate Test Selection

**Figure 4:**
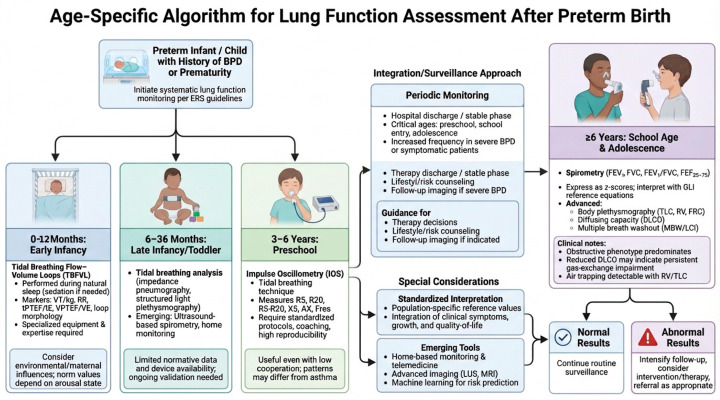
Age–specific algorithm for lung function assessment after preterm birth. Proposed clinical algorithm for structured pulmonary follow-up in preterm-born children, with or without bronchopulmonary dysplasia (BPD). The pathway emphasizes early initiation of lung function monitoring according to European Respiratory Society recommendations and age-adapted assessment strategies. In infancy (0–12 months), evaluation relies on tidal breathing flow-volume loop analysis. During late infancy and toddlerhood (1–3 years), tidal breathing-based techniques and emerging technologies may be applied. In preschool children (3–6 years), impulse oscillometry enables assessment of small airway function. From school age (≥6 years), spirometry represents the cornerstone of evaluation, complemented by advanced tests, including body plethysmography, diffusing capacity, and multiple breath washout, when indicated. The algorithm integrates periodic monitoring, phenotype-driven interpretation, and escalation of follow-up in case of abnormal findings, supporting individualized longitudinal surveillance into adolescence. BPD: bronchopulmonary dysplasia; ATS/ERS: American Thoracic Society/European Respiratory Society; TBFVL: tidal breathing flow-volume loop(s); VT/kg: tidal volume per kilogram; RR: respiratory rate; tPTEF/tE: time to peak tidal expiratory flow to expiratory time ratio; VPTEF/VE: ratio of volume to peak tidal expiratory flow to expiratory volume; IOS: impulse oscillometry (system); R5: respiratory system resistance at 5 Hz; R20: Resistance at 20 Hz; R5-R20: difference between resistance at 5 Hz and 20 Hz (reflecting peripheral airway resistance); X5: respiratory system reactance at 5 Hz; AX: area of reactance; Fres: resonant frequency; FEV_1_: forced expiratory volume in 1 s; FVC: forced vital capacity; FEV_1_/FVC: ratio of forced expiratory volume in 1 s to forced vital capacity; FEF25–75: forced expiratory flow between 25% and 75% of FVC; TLC: total lung capacity; RV: residual volume; FRC: functional residual capacity; DLCO: Diffusing Capacity of the Lung for Carbon Monoxide; MBW: Multiple Breath Washout; LCI: Lung Clearance Index. (Created with FigureLabs).

Systematic lung function monitoring in preterm-born children requires the selection of age-appropriate techniques based on developmental stage, feasibility, and local expertise (Figure 4). Because cooperation and technical reliability vary substantially across childhood, assessment strategies must evolve accordingly.

In the first year of life (0–12 months), lung function assessment relies primarily on non-volitional techniques. TBFVL analysis during natural sleep represents the most feasible approach, although it requires specialized equipment and trained personnel [67]. Toddlerhood (1–3 years) remains a particularly challenging period, characterized by limited cooperation and the transition from infant-specific to preschool methodologies. The availability of validated commercial devices is still restricted, and although emerging technologies show promise, their routine clinical implementation remains limited [46,47,48,49].

From approximately 3 years of age, IOS becomes increasingly feasible, with reported success rates exceeding 90% in many preschool populations when appropriate coaching and gamification strategies are used [68]. From 5–6 years onward, spirometry becomes the cornerstone of evaluation, with reproducibility improving progressively with age [38]. In selected cases, advanced testing (including body plethysmography, diffusing capacity, or multiple breath washout) may provide complementary physiological information.

In clinical practice, structured assessment algorithms integrated into multidisciplinary follow-up programs may enhance standardization, improve longitudinal comparability, and optimize resource allocation [38].

From a feasibility perspective, a tiered model may facilitate the translation of follow-up recommendations across different healthcare settings. At a minimum, centers with limited resources should ensure longitudinal clinical assessment, growth monitoring, pulse oximetry when indicated, and spirometry from school age onward, ideally integrated with review of symptom burden, exacerbations, and environmental exposures. Where available, a more comprehensive phenotyping approach may additionally include IOS in preschool children and body plethysmography, DLCO, or multiple breath washout in school-age children and adolescents with abnormal spirometry, persistent symptoms, or suspected peripheral airway or gas-exchange impairment. This resource-stratified perspective may broaden applicability without implying that all centers must implement the full testing panel at every visit [38].

### 7.2. Interpretation Frameworks

Interpretation of lung function in preterm-born children requires a comprehensive and standardized approach. Results should be expressed as z-scores using appropriate reference equations that account for anthropometric variables such as age, height, and sex. In some contexts, population-specific reference standards may be necessary, given variability related to ethnicity and measurement devices [69]. The lower limit of normal, typically defined as the 5th percentile or a z-score < −1.64, provides a statistically derived threshold for abnormality [56]. However, statistical definitions do not always correspond to clinical significance. In preterm-born populations, even values within the conventionally “normal” range may carry prognostic relevance, reflecting a reduced physiological reserve and potential susceptibility to accelerated functional decline [70].

Beyond threshold-based interpretation, clinicians should integrate patterns of abnormality (including obstructive versus restrictive defects, central versus peripheral airway involvement, and impaired gas exchange) thereby moving beyond a purely dichotomous classification toward a more physiologically informed assessment [71].

### 7.3. Integration into Longitudinal Care

The 2020 ERS guidelines recommend lung function monitoring in children with established BPD, although optimal frequency is not definitively defined [38]. A pragmatic schedule for clinical implementation may include an initial assessment at hospital discharge or once the infant is clinically stable, followed by reassessment during toddlerhood when clinically indicated, at preschool age, at school entry, and again during adolescence, with intermediate evaluations approximately every 2–3 years depending on symptoms, prior results, and local resources.

Closer surveillance may be warranted in children with severe BPD, persistent respiratory symptoms, recurrent exacerbations, or ongoing need for respiratory medications [38].

Clinical decision-making should integrate lung function findings within the broader clinical context, including symptom burden, health-related quality of life, exacerbation history, and growth patterns. Abnormal lung function in otherwise asymptomatic children may prompt counseling on modifiable risk factors (such as tobacco smoke exposure) and reinforcement of infection prevention strategies, alongside closer follow-up. Conversely, documented functional impairment in symptomatic patients may justify therapeutic trials and structured evaluation of treatment response [38].

### 7.4. Transition to Adult Care

A comprehensive life-course model of care should encompass a structured transition from pediatric to adult respiratory services, with particular emphasis on adolescents born extremely or very preterm. This transition is especially crucial for individuals with a history of moderate-to-severe bronchopulmonary dysplasia (BPD), persistent airflow limitation, abnormal diffusing capacity, or ongoing respiratory symptoms. During the transition, a concise summary should ideally include gestational age, neonatal respiratory history, BPD severity when applicable, major respiratory exposures, exacerbation and hospitalization history, peak lung function achieved during pediatric follow-up, longitudinal spirometric trends, evidence of prematurity-associated obstructive lung disease or other phenotypic traits, current treatments, and relevant imaging or gas-exchange findings. This information can facilitate continuity of care, mitigate misclassification as classical asthma alone, and enhance surveillance for early chronic obstructive pulmonary disease-like trajectories in adulthood [11,13].

## 8. Research Gaps, Emerging Technologies and Future Directions

Despite substantial advances in the understanding of respiratory outcomes after preterm birth, important gaps remain in both mechanistic knowledge and long-term clinical management. A major limitation in current research and practice is the lack of standardized, age-specific lung function protocols across centers [59,60]. Variability in timing of assessment, methodology, equipment, and reference equations contributes to heterogeneous reporting and complicates comparison across studies. The development of harmonized consensus guidelines addressing timing, techniques, quality control, and interpretation across pediatric age groups would improve reproducibility, facilitate multicenter collaboration, and strengthen the evidence base for long-term follow-up strategies [38].

At the biological level, the mechanisms underlying persistent lung function impairment remain incompletely elucidated [61]. The frequent dissociation between structural abnormalities on imaging, physiological impairment, and clinical symptoms suggests a multifactorial pathophysiology involving airway, parenchymal, vascular, and immune components [10]. Integrative multi-omic approaches (combining transcriptomic, proteomic, and metabolomic profiling with longitudinal functional data) may help identify molecular signatures associated with specific trajectories and distinguish delayed maturation from progressive remodeling processes [72]. Such integrative strategies may ultimately bridge developmental biology with individualized risk prediction and precision follow-up.

The long-term impact of neonatal interventions also warrants further investigation. Although strategies such as surfactant therapy, caffeine, and vitamin A supplementation reduce the incidence or severity of BPD, their influence on adult lung function trajectories remains uncertain [73]. Prospective trials evaluating post-discharge interventions and airway-directed therapies are needed to determine whether early modulation of lung injury can alter established impairment.

In parallel, emerging technologies are expanding the tools available for phenotyping and longitudinal surveillance. Advanced imaging modalities provide complementary structural information beyond physiological testing. Quantitative computed tomography can characterize emphysematous changes, bronchial wall thickening, mosaic attenuation, and air trapping, offering insight into structural correlates of airflow limitation; however, cumulative radiation exposure limits routine pediatric use [74]. Magnetic resonance imaging, including techniques assessing regional ventilation and perfusion, offers radiation-free evaluation and has demonstrated feasibility in infants and children with BPD [75]. Lung ultrasound has shown value in the neonatal period for predicting and monitoring BPD, although its role in long-term structural assessment remains to be defined [76].

Digital health tools and remote monitoring strategies may further enhance longitudinal care. Portable spirometry, home pulse oximetry, and wearable sensors could enable continuous or intermittent data collection in children at risk for respiratory complications. Integration with electronic health records and clinical decision-support systems may support earlier detection of deterioration and more individualized follow-up strategies [77]. Nevertheless, challenges related to data quality, adherence, and standardization require careful consideration.

Finally, predictive modeling represents a promising frontier. Machine learning approaches integrating clinical, physiological, imaging, and environmental variables may improve risk stratification and help forecast long-term respiratory trajectories [78,79]. However, robust external validation and harmonized multicenter datasets are essential before these tools can be safely translated into routine practice.

Future research should therefore prioritize large, longitudinal cohorts incorporating detailed prenatal and postnatal exposure data together with serial lung function assessments at predefined developmental stages [12]. Multicenter collaborations with standardized protocols and centralized data repositories will be critical to advancing from descriptive epidemiology toward precision follow-up. Early identification of high-risk profiles may ultimately enable tailored surveillance, targeted preventive strategies, and optimized therapeutic decision-making, thereby improving lifelong respiratory outcomes for individuals born preterm.

## 9. Conclusions

Age-specific lung function assessment in preterm-born children requires a structured and developmentally tailored approach that evolves with cooperation, physiological maturation, and clinical needs. From tidal breathing measurements in infancy to IOS in preschool age and spirometry with advanced testing in school age children and adolescents, each modality provides complementary insight into respiratory health across the life course.

Accumulating evidence consistently demonstrates that preterm birth is associated with persistent alterations in lung function extending into adolescence and likely adulthood. Importantly, respiratory impairment is not confined to individuals with BPD, as prematurity per se contributes substantially to long-term functional vulnerability. These findings support a continuum model of chronic lung disease of prematurity rather than a dichotomous classification based solely on neonatal oxygen requirement.

Systematic lung function monitoring enables early identification of at-risk individuals, facilitates risk stratification, and may guide preventive and therapeutic strategies. However, effective implementation requires standardized protocols, age-appropriate methodologies, trained personnel, and interpretation frameworks based on robust reference equations and clinical context.

Future efforts should prioritize harmonization of assessment strategies, clarification of underlying pathophysiological mechanisms, identification of modifiable risk factors, and development of interventions aimed at optimizing lung growth trajectories in this vulnerable population. A life-course perspective integrating neonatal history with longitudinal functional evaluation is essential to improve long-term respiratory outcomes for preterm-born children.

## Figures and Tables

**Table 1:** Developmentally tailored lung function assessment in preterm-born children. Overview of age-specific lung function techniques, principal physiological parameters, methodological strengths and limitations, and evidence of abnormalities reported in preterm-born populations. Assessment feasibility and interpretability evolve across developmental stages, highlighting the importance of structured longitudinal follow-up.

Age Group	Main Techniques	Key Parameters	Strengths	Limitations	Evidence
Infancy	TBFVL RVRTC	VT/kg, RR, tPTEF/tE, VmaxFRC	Non-volitional; early phenotypic stratification	Requires expertise; sedation (RVRTC); limited normative data	Early abnormalities associated with BPD severity and later respiratory morbidity
Toddlerhood	Tidal breathing techniques; emerging technologies (ultrasound spirometry, impedance pneumography, structured light plethysmography)	Expiratory variability index; ventilation efficiency indices	Low-cooperation feasibility	Limited validation; lack of standardized reference values	Promising but insufficient longitudinal data
Preschool Age	IOS MBW	R5, R20, R5–R20, X5, AX, LCI	Quiet breathing; sensitive to small airway dysfunction	Device variability; reference equation heterogeneity	Persistent peripheral airway dysfunction
School Age and Adolescence	Spirometry Body plethysmography; DLCO MBW	FEV_1_, FVC, FEV_1_/FVC, FEF25–75, TLC, RV/TLC, DLCO, LCI	Standardized interpretation (GLI z-scores); full physiological profiling	Requires cooperation; specialized equipment for advanced tests	Persistent airflow limitation, reduced DLCO, risk of COPD-like trajectories

TBFVL: tidal breathing flow-volume loop; RVRTC: raised volume rapid thoracoabdominal compression; VT/kg: tidal volume per kilogram; RR: respiratory rate; tPTEF/tE: time to peak tidal expiratory flow to expiratory time ratio; VmaxFRC: maximal expiratory flow at functional residual capacity; IOS: Impulse Oscillometry (System); MBW: Multiple Breath Washout; R5: respiratory system resistance at 5 Hz; R20: Resistance at 20 Hz; R5–R20: difference between resistance at 5 Hz and 20 Hz (reflecting peripheral airway resistance); X5: respiratory system reactance at 5 Hz; AX: area of reactance; LCI: Lung Clearance Index; DLCO: Diffusing capacity of the lung for carbon monoxide; FEV_1_: forced expiratory volume in 1 s; FVC: forced vital capacity; FEV_1_/FVC: ratio of forced expiratory volume in 1 s to forced vital capacity (Tiffeneau index); FEF25–75: forced expiratory flow between 25% and 75% of FVC; TLC: Total Lung Capacity; RV/TLC: Residual Volume-to-Total Lung Capacity ratio (air trapping/hyperinflation index); COPD: Chronic Obstructive Pulmonary Disease.

## Data Availability

No new data were created or analyzed in this study.

## Abbreviations

The following abbreviations are used in this manuscript:

AX	Area of reactance
ATS	American Thoracic Society
BPD	Bronchopulmonary dysplasia
COPD	Chronic obstructive pulmonary disease
DLCO	Diffusing capacity of the lung for carbon monoxide
ERS	European Respiratory Society
FEF25–75	Forced expiratory flow between 25% and 75% of forced vital capacity
FEV_1_	Forced expiratory volume in one second
FRC	Functional residual capacity
Fres	Resonant frequency
FVC	Forced vital capacity
GLI	Global Lung Function Initiative
IOS	Impulse Oscillometry System
LCI	Lung Clearance Index
MBW	Multiple Breath Washout
PEF	Peak expiratory flow
POLD	Prematurity-associated obstructive lung disease
QC	Quality control
R5	Respiratory system resistance at 5 Hz
R20	Respiratory system resistance at 20 Hz
R5-R20	Difference between respiratory resistance at 5 Hz and 20 Hz (index of peripheral airway resistance)
RR	Respiratory rate
RV	Residual volume
RV/TLC	Residual volume-to-total lung capacity ratio
RVRTC	Raised-volume rapid thoracoabdominal compression
TBFVL	Tidal breathing flow-volume loop
TLC	Total lung capacity
tPTEF/tE	Ratio of time to peak tidal expiratory flow to total expiratory time
VPTEF/VE	Ratio of volume to peak tidal expiratory flow to expiratory volume
VT/kg	Tidal volume corrected for body weight
X5	Respiratory system reactance at 5 Hz
